# Faecal glucocorticoid metabolites and body temperature in Australian merino ewes (*Ovis aries*) during summer artificial insemination (AI) program

**DOI:** 10.1371/journal.pone.0191961

**Published:** 2018-01-30

**Authors:** Edward Narayan, Gregory Sawyer, Simone Parisella

**Affiliations:** 1 School of Science and Health, Western Sydney University, Locked Bag 1797, Penrith, New South Wales, Australia; 2 Graham Centre for Agricultural Innovation, School of Animal and Veterinary Sciences, Charles Sturt University, Locked Bag 588, Wagga Wagga, New South Wales, Australia; Centre for Cellular and Molecular Biology, INDIA

## Abstract

Reproductive wastage is a key issue for sheep producers, both regionally and globally. The reproductive output of farm animals can be influenced by physiological and environmental factors. Rapid and reliable quantification of physiological stress can provide a useful tool for designing and testing on-farm management interventions to improve farm animal welfare and productivity. In this study, we quantified physiological stress non-invasively using faecal glucocorticoid metabolites-FGMs analysis and body temperature measurements of 15 superovulated donor merino ewes (*Ovis aries*) during participation in artificial insemination (AI) program conducted during 2015/2016 Australian summer. We hypothesized that low percentage transferable embryos in donor merino ewes will be associated positively with higher body temperature and/or higher FGMs in these ewes. Temperature humidity index (THI) was calculated and found within high thermal stress range during the two AI trials. Overall, results showed none of the factors (ewe ID, AI trial no., THI or FGMs) were significant for reduced percentage transferrable embryos, except ewe body temperature was highly significant (p = 0.014). Within AI trial comparisons showed significant positive associations between higher FGMs and body temperature with reduced transferrable embryos. These results suggest that Australian merino ewes participating in summer AI trials can experience physiological stress. Prolonged activation of the stress endocrine response and high body temperature (e.g. ensued from heat stress) could impact on ewe reproductive output. Therefore, future research should apply minimally invasive physiological tools to gather baseline information on physiological stress in merino sheep to enable the development of new farm-friendly methods of managing stress.

## Introduction

Sheep production systems in arid landscapes (e.g. regional Australia) are frequently exposed to high ambient temperature or ‘heat waves’ during the summer period [1, 2]. Review work of [3] explains how key biological functions (e.g. enzyme-balance, hormone secretions and feed intake efficiency) are affected by exposure to heat stress in sheep, which results in reduced reproductive function. Ewes exposed to ambient temperature (in an insulated room in which the temperature was maintained constantly at 32 °C with a variation of not more than l~ and with the relative humidity maintained at 60–65%), within 7 days of conception tend to produce more abnormal and retarded embryos, than non-heat stressed ewes [4]. Thermal or heat stress occurs when the environment acts to drive core body temperature above its normal range [5] and it is associated with hyperthermia [5, 6]. Stress caused by an elevation in body temperature in sheep is known to adversely affect embryo survival, implantation and fertility [7, 8], however, to our knowledge, the relationships between body temperature, physiological stress and percentage embryo transferable has not been tested under field conditions in Australian merino sheep.

Thermal stress in sheep has also been shown to negatively influence superovulation response and the rate of transferable embryos [9] during artificial insemination programs, suggesting that thermal stress ensued from extreme climate can affect sheep reproduction. [3] and others [2] have highlighted that further research on the physiological mechanisms of heat stress impacts is needed so that innovative methods of tackling the issue of global climate change on livestock production systems can be developed. This research acknowledges [9] for early research into this field, however the Australian Merino is a far superior merino sheep with outstanding attributes for greasy wool characteristics, <1% dark and medullated fibre content. The Bharat sheep is a composite breed of vast genetic makeup—unlike the Australian Merino which is a purer breed of Merino sheep.

A quantitative measurement of stress is *via* glucocorticoid levels in blood or excreta [10]. Faecal glucocorticoid metabolites (FGMs) evaluation is gaining popularity as a non-invasive index of physiological stress in livestock animals [11]. It is the measurement of FGMs present in faeces collected from the animal, which reflects the amount of cortisol present in blood prior to excretion and the hypothalamo-pituitary-adrenal (HPA) axis activity [12].

Glucocorticoids also provide a physiological index to heat stress response in animals, and other steroidal hormones such as oestradiol and progesterone can also be reduced due to heat stress [2]. The researchers explained that the activation of the HPA-axis and secretion of glucocorticoids is associated with the hypothalamic release of ACTH due to thermoreceptor activation. Furthermore, glucocorticoids provide necessary physiological changes acting as vasodilators to enable heat dissipation, also increasing metabolic activity (e.g. proteolysis and lipolysis) to off-set energy reduction due to reduced intake [2].

Thus, we tested the hypothesis that physiological measures (FGMs and body temperature) will be positively associated with a reduction in percentage transferrable embryos in donor Australian merino ewes within artificial insemination programs done within one Australian summer period of 2015/2016.

## Material and methods

All research samples were obtained within standard husbandry procedures of the client and biological samples were registered through the Veterinary Diagnostic Laboratory at Charles Sturt University. Charles Sturt University (CSU) Animal Care and Ethics Committee specifically approved this study (Protocol number: A16070).

### Animals and animal handling

All sheep used in this study were obtained and assessed at the Roseville Park Merino Stud, Dubbo, NSW 2830, Australia, and were part of their ongoing artificial insemination program. Fifteen non-pregnant merino ewes aged between 2 and 6 years old were individually assessed and selected for soundness to participate in the embryo transfer (ET) program. Information on age of each ewe and relevant physiological and environmental data (S1 Appendix).

Only superovulated donor ewes (those producing a large number of oocytes at one time) were used for examination in this study. All sheep remained at the Roseville Park Merino Stud property for the duration of the study, where all AI and ET procedures, sampling and data collection took place. The donor ewes were used twice for AI program in summer period of both November 2015 and February 2016. We also compared results obtained between the two AI trials. All handling of sheep was done by trained personnel. Data and sample collection (faeces and rectal temperature) of the 15 ewe sheep was performed by an experienced sheep handler. No blood samples were taken due to precautionary measures as these were commercially valuable ewes so faeces were collected to index physiological stress non-invasively (FGMs). All AI and ET procedures that were carried out on sheep in this study were done by qualified sheep AI/ET technicians. All ewes were carefully monitored for any signs of stress for 3 days post sampling.

### Body temperature and faecal sample collection

Rectal temperature and faecal samples were obtained from all donor merino ewes (AI Trial 1—November 20^th^, 2015 and AI Trial 2- February 5^th^, 2016). Fresh faeces were collected from donor ewes on the day of AI (see section 2.3 AI procedure) so that the sample represented basal FGMs levels. Adrenocortical stimulation in sheep has been demonstrated using ACTH challenge showing that cortisol clearance from blood to faeces occurs in 10 hours [13]. Thus the faeces collected prior to AI were going to be absent of any stressors associated with yarding and handling sheep.

Rectal temperature was taken twice from each donor ewe on the day of AI (see section 2.3 AI procedure), firstly in the morning when ewes were brought into the paddock and secondly in the afternoon 30 mins after the AI procedure. Prior to first sample collection, ewes were brought into pens from the open paddocks and individually assessed and condition scored to ensure each animal was of sound condition and fit to participate in the breeding program. One at a time, ewes were gently restrained into an upright position where a rectal thermometer was placed into the anus of the ewe and rectal temperature was recorded. A fresh faecal sample was collected from each ewe manually from the rectum (assessment and sampling took < 3 mins per ewe). Faecal samples were placed into labelled zip lock bags, where they were stored in a cool box on ice until delivery to the laboratory. All faecal samples were stored at -80 °C until analysis.

After samples were collected, ewes were released and left to recover in a pen, where the ewes were monitored for any signs of stress. The second rectal temperature was taken in the afternoon on the day of AI within 30 mins of the AI procedure.

### Artificial insemination (AI) and embryo transfer

The current standard AI and ET protocol in Australia based on AllStock Artificial Breeding Services (www.allstock.com.au) was implemented. Donor ewes were artificially inseminated *via* laparoscopy, with frozen semen that had been thawed on the day of insemination. All laparoscopic AI procedures conducted on the donor ewes were performed by the same qualified AI technician. On the day of AI, donor ewes were sedated with 0.05 mg/kg of Zylazil injection 20 minutes prior to the AI procedure. Donor ewes were then artificially inseminated via laparoscopy, with fresh and frozen semen. Faecal samples were collected prior to AI while rectal temperature was recorded twice (immediately before sedation and 30 min post AI). All laparoscopic AI procedures conducted on donor ewes in this study were performed by a qualified veterinary surgeon.

On day of embryo transfer, donor and recipient ewes had feed and water removed. ET was performed from donor and to the recipient ewes. Both donor and recipient ewes were injected with 0.05 mg/kg of Zylazil for sedation 20 minutes before the procedure. Flushing of donor ewes and embryo transfer into recipient ewes was performed by a qualified veterinary surgeon. All embryos were assessed by a qualified embryologist to determine which embryos were suitable for transfer into recipient ewes. The percentage transferable embryos (%) was determined for each superovulated donor ewe based on the number of embryos produced and the number of embryos fertilized (S1 Appendix).

### Faecal cortisol metabolite enzyme-immunoassay (EIA)

**Fig 1:**
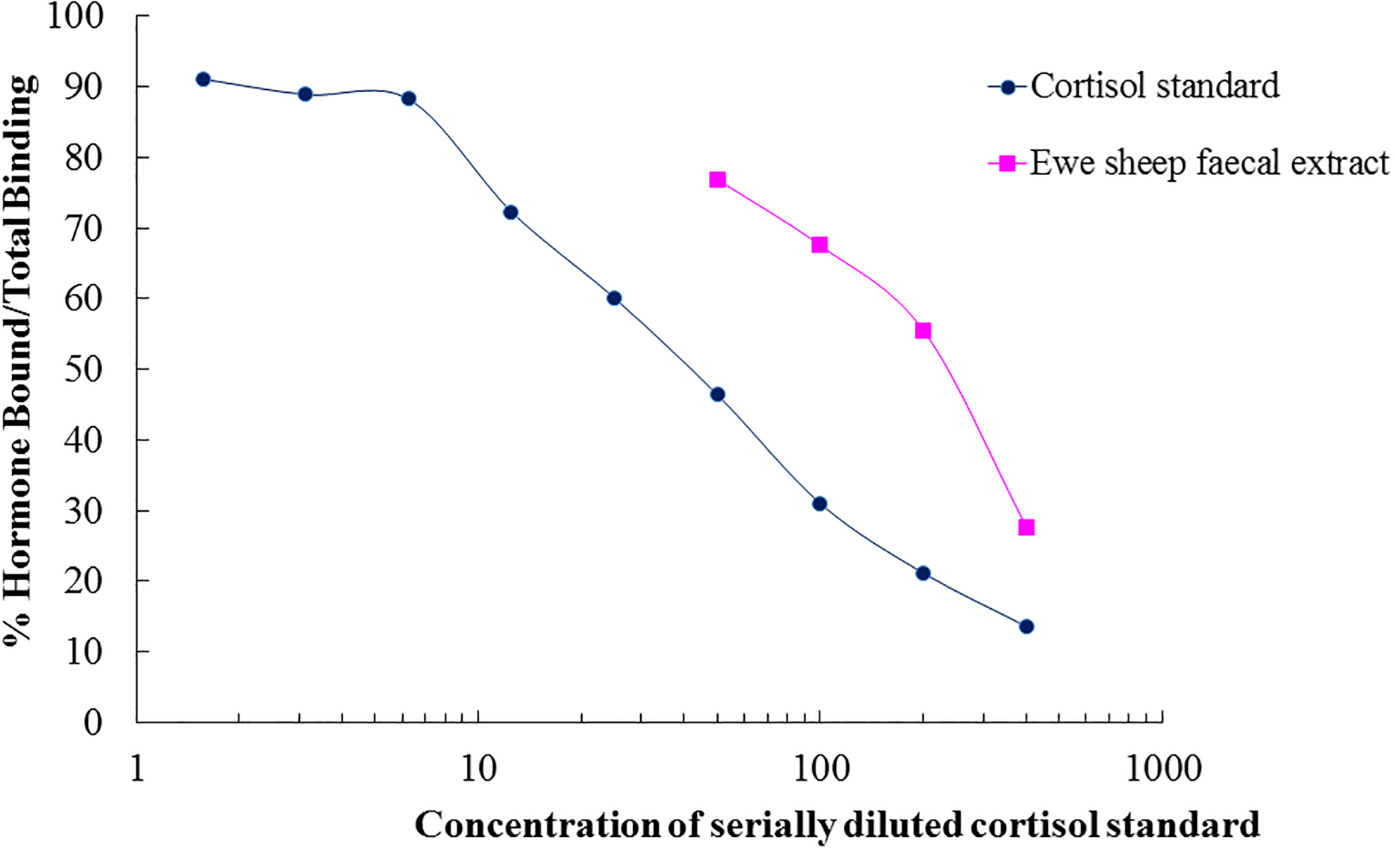
Parallelism displacement curve for cortisol standard and merino ewe faecal extracts run in serial dilutions. The y-axis demonstrates the percentage of hormone bound/total binding and the x-axis demonstrates the concentration of cortisol of standards and pooled faecal extracts. The 30% and 70% binding points determined the dilution factors for the high- and low-binding internal controls, respectively. 50% binding point determined the dilution factor for assaying faecal extracts to quantify faecal cortisol metabolites.

Faecal cortisol metabolites concentration was determined by EIA analysis using polyclonal anticortisol antiserum (R4866 –supplier Coralie Munro, UC Davies, Davies, California) diluted in ELISA coating buffer (Carbonate-Bicarbonate Buffer capsule Sigma C-3041 and 100 mL Milli-Q water, pH 9.6), with a working dilution of 1:15,000. This was followed by reactivity with Horseradish Peroxidase (HRP) conjugated cortisol label (UC Davis, Davis, California) diluted 1:80,000, and cortisol standards diluted serially (1.56 – 400pg well^-1^). Nunc Maxi-Sorp^™^ plates (96 wells) were coated with 50 μL cortisol antibody solution and incubated for a minimum of 12 hours at 4 °C. Laboratory validation was achieved using parallelism. Cortisol standards and faecal extract pool were prepared serially using the EIA buffer and loaded on the same plate. The 50% binding point on the parallelism curve determined the assay dilution factor and the 30% and 70% binding points determined the dilution factors for the high and low binding points respectively (Fig 1).

For all assays, 50 μL of standard, control or diluted faecal extract were added to each well, followed by 50 μL of the diluted cortisol HRP. Each plate was loaded in under 10 minutes. Plates were covered with acetate plate sealer and incubated at room temperature for 2 hours. After incubation, plates were washed 4 times using an automated plate washer (ELx50, BioTeck^™^) with phosphate-buffered saline solution (0.05% Tween 20) and blotted on paper towel to remove excess wash solution.

Substrate buffer (TMB) was prepared by combining 1 μL 30% H_2_O_2_, 75 μL 1% tetramethylbenzidine and 7.425 μL 0.1 M acetate citrate acid buffer, pH 6.0, per plate. The TMB substrate was added to each well that contained standard, control and diluted faecal extract at 50 μL, to generate colour change. The plates were covered with an acetate plate sealer and left to incubate at room temperature for 15 minutes. The reaction was stopped with 50 μL of Stop solution (0.5 M H_2_SO_4_ and Milli-Q water) added to all wells in the Nunc Maxi-Sorp^™^ plates. To determine the concentration of faecal cortisol metabolite in each sample, plates were read at 450nm (reference 630nm) on an ELx800 (BioTek^™^) microplate reader.

### Local climatic data

Climate data for Dubbo was obtained from the Bureau of Meteorology, Government of Australia [14].

### Statistical analysis

Statistical analysis and graphs was done using EXCEL and SYSTAT version 13.0. The temperature humidity index (THI) was used to index heat stress experienced by the sheep. We calculated THI using the formula THI equals 0.8*T + RH*(T-14.4) + 46.4 where T = ambient or dry-bulb temperature in °C and RH = relative humidity expressed as a proportion i.e. 80% humidity is expressed as 0.80 (Source: http://www.veterinaryhandbook.com.au/ContentSection.aspx?id=51). Mean monthly THI and other climatic data between the two AI trials were compared using t-test. See S1 Appendix for the climatic data and THI calculation.

Statistical analysis was done to test the hypothesis that (1) physiological measures (FGMs and ewe body temperature) will be positively associated with reduction in percentage transferrable embryos in merino donor ewes and with (2) increasing ambient temperature in artificial insemination program done in Australian summer. Firstly, we analysed the overall effects of the physiological variables on percentage transferrable embryos using a Generalised Linear Model ANOVA with AI trial, ewe ID, FGMs and rectal temperature post AI as the factors and percentage transferrable embryos as the dependant variable.

We also used a least squares regression model to determine the level of significance of all factors (THI, FGMs, AI trial no., and body temperature) on percentage transferred embryos. Secondly, we determined the associations within each AI trial using linear regression and obtained regression coefficient (r) and p value. Level of significance for all statistical analysis was p < 0.05.

## Results

### Comparison of climatic variables between AI trials

The mean monthly THI for AI trial 1 was significantly higher than the mean monthly THI for AI trial 2 (t = 2.42, df = 57, p = 0.009) [Table 1]. Ambient temperature at the time of AI trial 1 was 39.9 °C and AI trial 2 was 28.5 °C. Mean monthly (max-min) ambient temperature and relative humidity between the two AI trial periods were also significantly different (p < 0.05 for all comparisons) [Table 1].

**Table 1:** Physiological and climatic data recorded during AI trial 1 and 2.

Climatic and physiological variables	AI Trial 1, 20^th^ November 2015	AI Trial 2, 5^th^ February 2016	P value
Morning Rectal Temp (°C)	39.15 + 0.09 (38.40–39.80)	39.31 + 0.08 (38.70–39.70)	Not significant (n.s)
Afternoon Rectal Temp (°C)	39.49 ± 0.08 (39.10–40.10)	39.27 ± 0.07 (38.70–39.40)	Weak significance (p = 0.057)
Unit Change in Rectal Temp (°C)	+0.35 + 0.108 (-0.2–0.7)	-0.03 + 0.07 (-0.3–0.80)	p < 0.05
Transferable Embryos (%)	45.72 ± 8.83 (0–90%)	52.33 ± 10.83 (0–100%)	p < 0.05
FCM (ng/g dry weight)	25.81 ± 4.28 (8.38–64.75)	21.71 ± 2.98 (11.20–40.18)	p < 0.05
Maximum ambient temp at AI (°C)	39.9	28.5	
Mean monthly max. temp prior to AI (°C)	30.42 ± 0.64 (28.1–39.9)	33.26 ± 0.57 (28.5–39.1)	p < 0.05
Mean monthly min. temp prior to AI (°C)	14.08 ± 0.50 (8.9–18.1)	16.82 ± 0.53 (9.7–20.3)	p < 0.05
Mean monthly max. relative humidity prior to AI	88.40 ± 2.45 (70–100)	74.66 ± 2.29 (37–91)	p < 0.05
Mean monthly min. relative humidity prior to AI	34.30 + 2.42 (16–58)	21.00 + 2.25 (1–46)	p < 0.05
Temp. Humidity Index (THI)	84.82 + 1.08 (75–101)	79.66 + 2.26 (59–93)	p < 0.05

### Overall influence of climatic and physiological variables on transferable embryos

As shown in Table 1, the percentage transferrable embryos, body temperature and FGMs levels were significantly different between both AI trials. The overall Generalised Linear Model ANOVA result showed that all factors were non-significant (ewe ID, AI trial no., FGMs and body temperature) with percentage transferrable embryos as dependant variable, only FGMs and body temperature were weakly non-significant (p = 0.058 and 0.071 respectively). Post-hoc comparison showed that the interaction/combined effect of FGMs and rectal temperature on percentage transferrable embryos was statistically significant (p = 0.045), however the interaction between ewe ID and AI trial was not statistically significant (p > 0.05). The overall least squares regression analysis determined that for all factors (AI trial no., THI and FGMs) none were significant factors for reduced percentage transferrable embryos. Only ewe body temperature was highly significant (p = 0.014).

### Influence of physiological variables on transferable embryos within each AI trial

During AI trial 1 (Nov 2015), only 14 ewes were used for the trial (S1 Appendix). 7 out of the 14 ewes (53.84%) yielded greater that 70% transferable embryos. All ewes (except one) recorded at least +0.1 °C change in body temperature after the AI procedure. Change in body temperature ranged from– 0.1 to + 0.7 °C (S1 Appendix).

**Fig 2:**
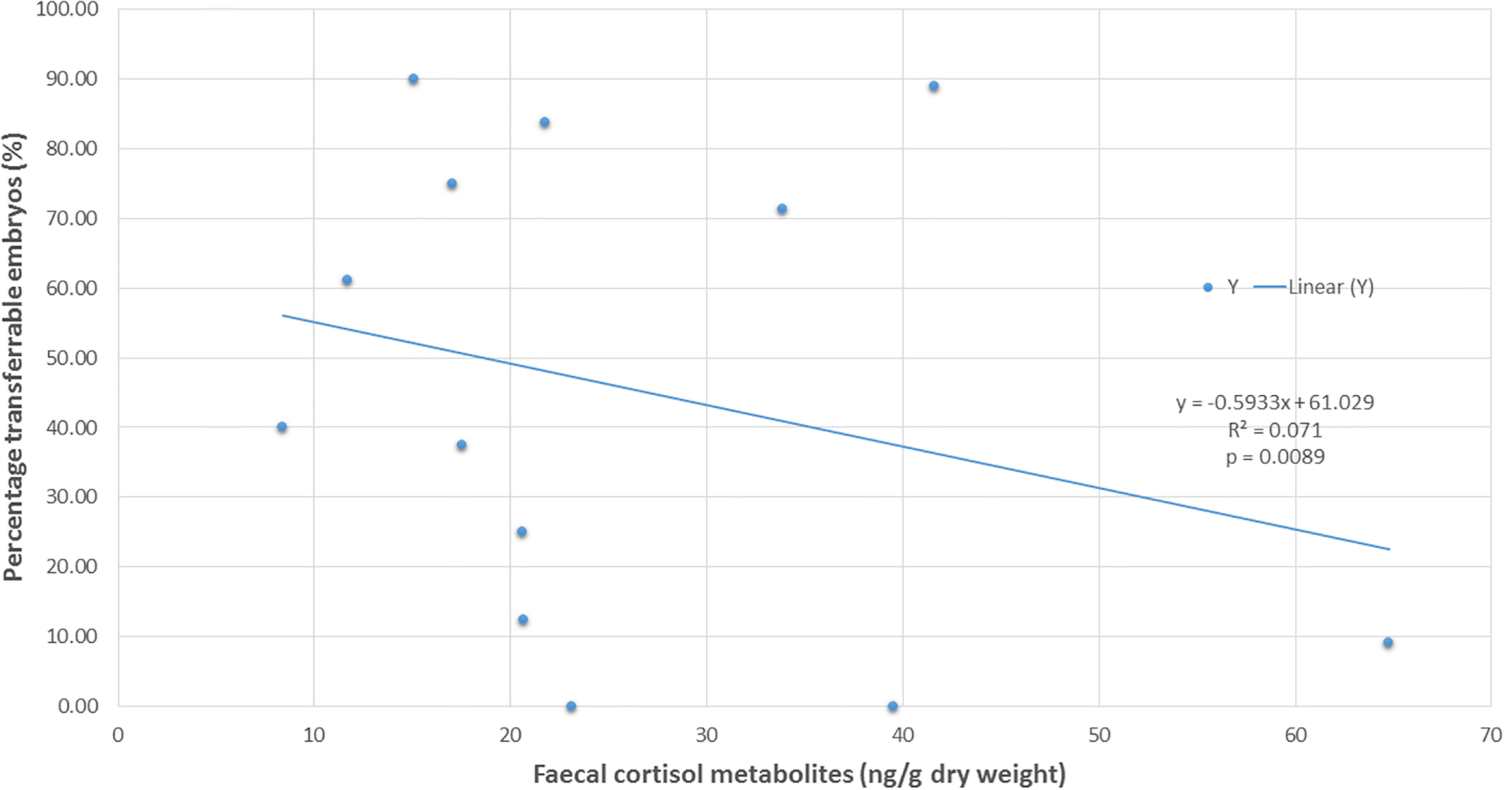
Relationship between faecal cortisol metabolites and percentage transferrable embryos: AI trial 1. Linear regression between FCM (ng/g dry weight) obtained from faecal extracts of donor merino ewes (n = 14) taken on the day of AI trial 1 on 20 November 2015, and percentage transferable embryos (%).

Within AI trial 1, a significant positive association was found between FGMs levels and reduction in percentage of transferable embryos (n = 13 ewes with recorded FCM data from AI trial 1) (r = +0.266, p < 0.05; Fig 2).

**Fig 3:**
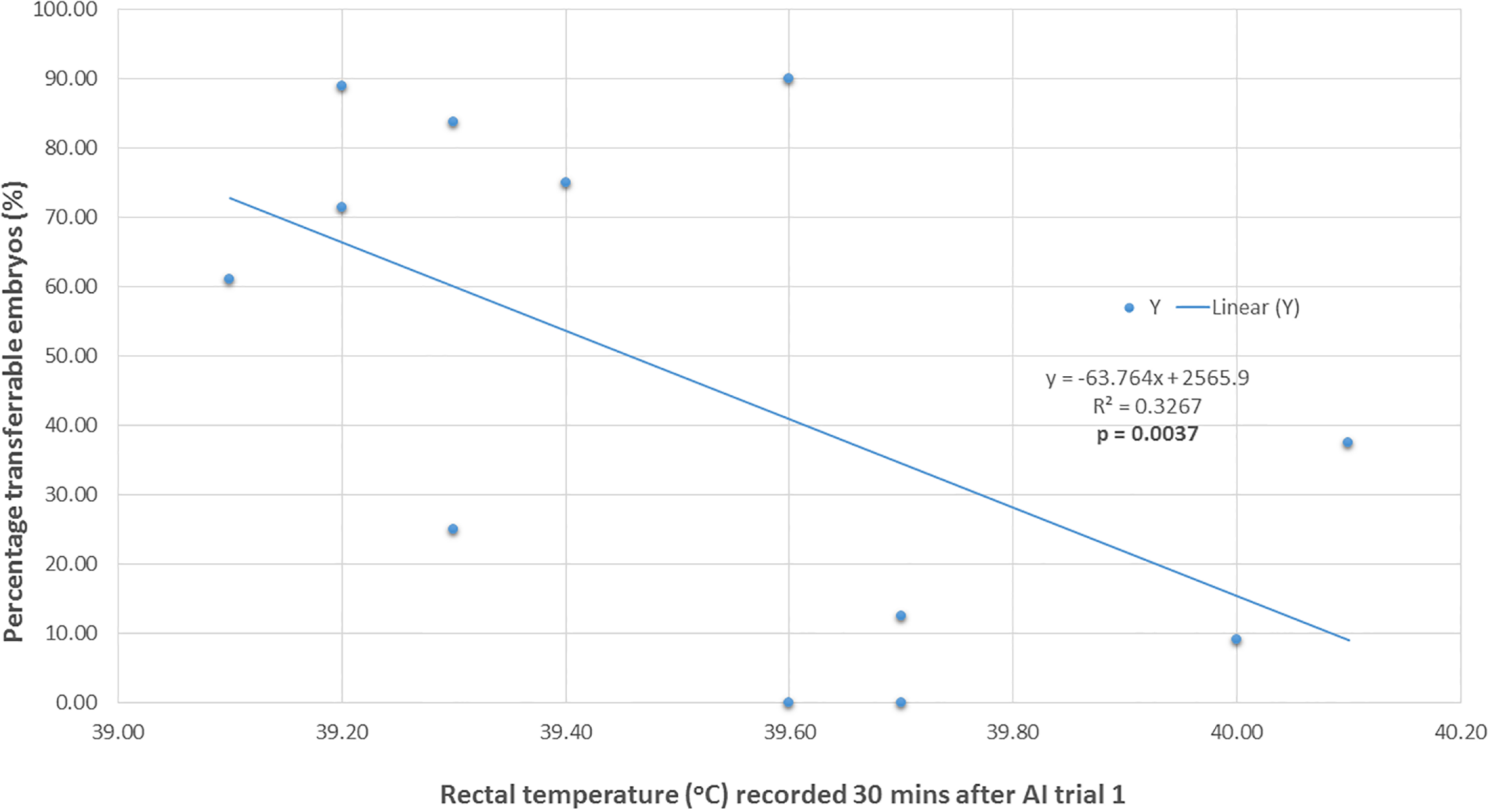
Relationship between percentage transferrable embryos and rectal temperature: AI trial 1. Linear regression between the percentage of transferable embryos (%) and rectal temperature (°C) of superovulated donor ewes (n = 15) recorded 30 mins post AI procedure on 20 November 2015.

Within AI trial 1, a significant positive association was also found between body temperature of ewes (recorded 30 mins after the AI) and reduction in percentage of transferable embryos (n = 14 ewes with temperature data from AI trail 1) (r = + 0.214, p < 0.05; Fig 3).

AI trial 2 (Feb 2016), 15 ewes were used for the trial (S1 Appendix). Only 6 out of 15 ewes (40%) yielded greater that 70% transferable embryos. A number of ewes recorded a drop in body temperature after the AI procedure (n = 9) while others showed change in body temperature from– 0.3 to + 0.8 °C (S1 Appendix).

**Fig 4:**
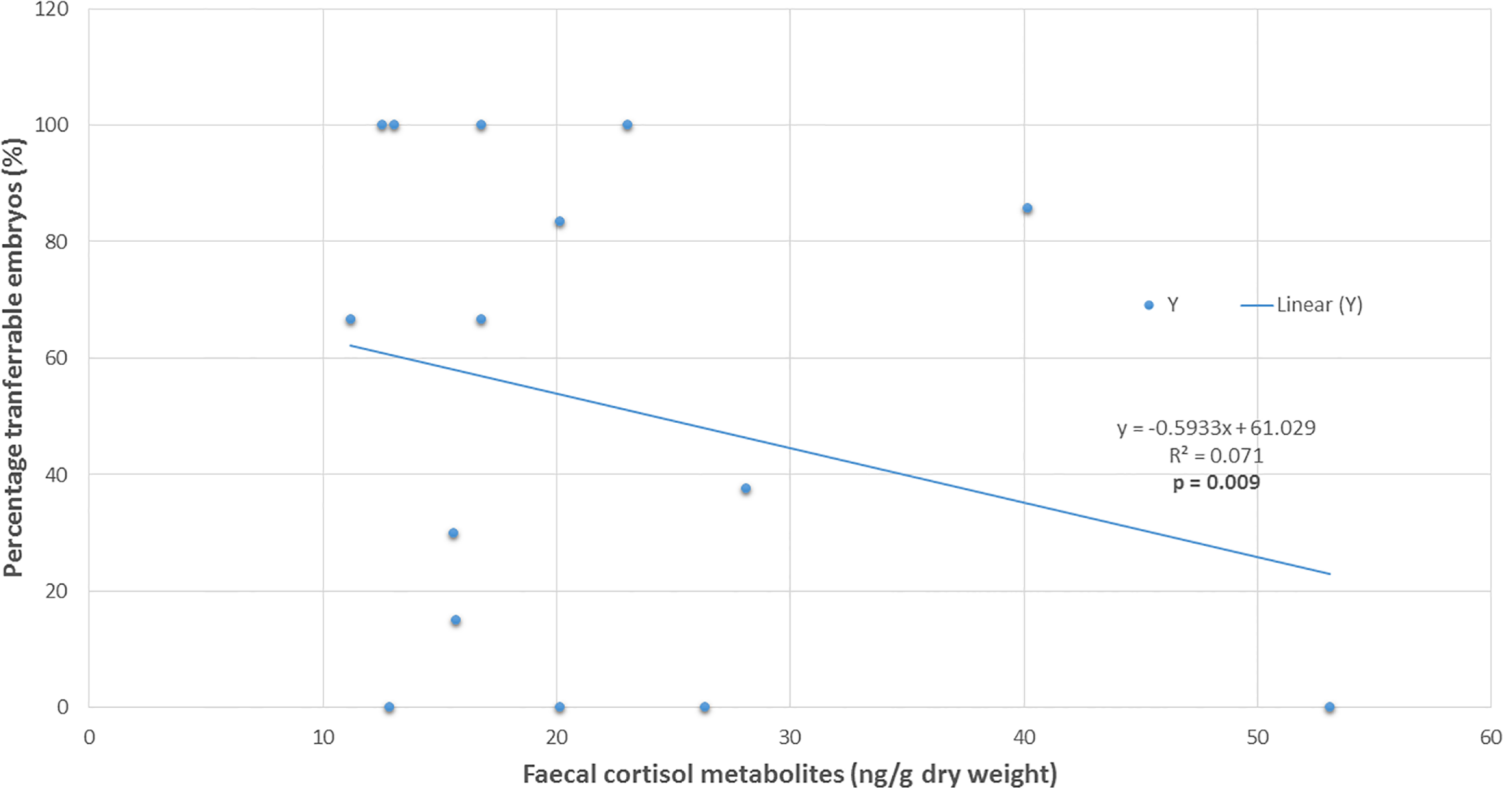
Relationship between faecal cortisol metabolites and percentage transferrable embryos: AI trail 2. Linear regression between FCM (ng/g dry weight) obtained from faecal extracts of donor merino ewes (n = 15) taken on the day of AI trial 2 on on 5 February 2016, and percentage transferable embryos (%).

Within AI trial 2, a significant positive association was found between faecal cortisol metabolite levels and reduction in percentage transferable embryos (n = 15 ewes with recorded FCM data from AI trail 2) (r = +0.257, p < 0.05; Fig 4).

**Fig 5:**
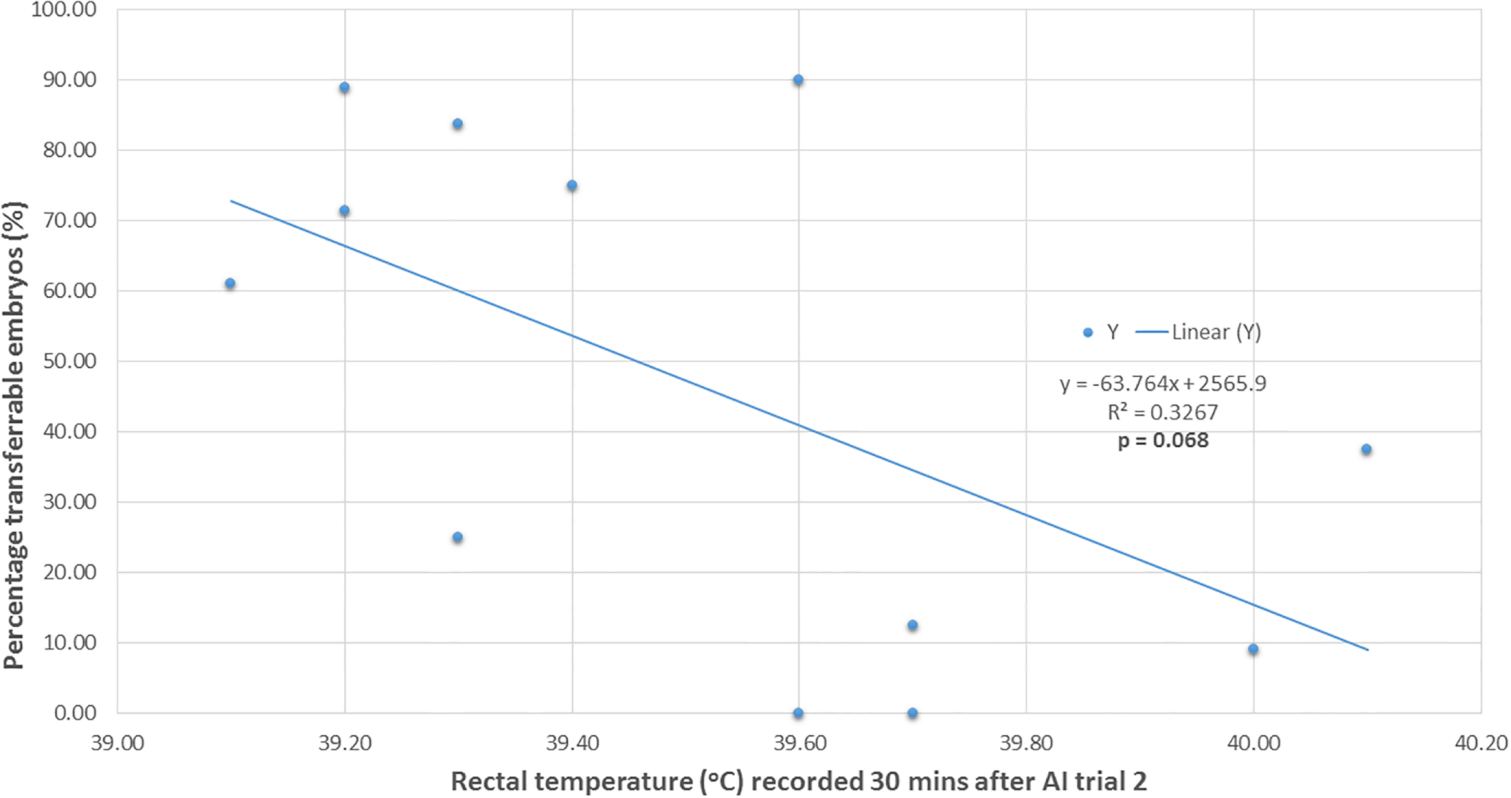
Relationship between percentage transferrable embryos and rectal temperature: AI trail 2. Linear regression between percentage of transferable embryos (%) and rectal temperature (°C) of superovulated donor ewes (n = 15) at the time of AI on 5 February 2016.

Within AI trial 2, a weak positive association was also found between body temperature of ewes (recorded 30 mins after the AI trial) and reduction in percentage of transferable embryos (n = 15 ewes with temperature data from AI trail 2) (r = +0.482, p = 0.068; Fig 5).

A weak positive association between rectal temperature and FCMs of the donor ewes during AI trial 1 (r = +0.482, p = 0.064), however, there was no significant association between rectal temperature and FCMs of the donor ewes during AI trial 2 (r = +0.09, p = 0.75).

## Discussion

In this study, we tested the physiological response variables (faecal glucocorticoid metabolites and body temperature) in superovulated donor Australian merino ewes during participation in summer AI trials. Overall results showed that out of all climatic and physiological factors, only ewe body temperature was statistically significant for reduced percentage transferrable embryos. The interaction between body temperature and FGMs was also significant. Temperature humidity index was within thermal stress range during the summer AI trials, however results did not indicate a statistically significant influence of heat stress (THI) on embryo loss. To our knowledge there has been no previous study based on physiological stress evaluation of Australian merino ewe sheep participating in summer AI program therefore our data provides useful baseline information for monitoring AI programs. Our results provide further support to earlier study that was conducted on Bharat merino ewes by [9], which showed that exposure to thermal stress significantly reduced quality embryo yield in the Bharat merino ewes. Our study has shown that physiological stress through elevated FGMs and body temperature could be impacting the reproductive output Australian merino ewes and these physiological changes could be directly/indirectly related to summer heat stress.

The Australian merino sheep breeding industry is heavily reliant on the breeding efficacy of its ewes in order to optimize lambing percentage and reproductive success to maintain a profitable business. The merino ewe is the driving force of the wool and sheep meat industry both locally and globally as it provides both merino and cross bred lambs to the industry, and furthermore passes on valuable genetics on to the next generation. In recent years, there has been a significant decline in ewe conception rates in Australia following breeding programs: artificial insemination (AI) and embryo transfer (ET) [15]. Recent data obtained from the Australian Sheep CRC Information Nucleus Flock from 2007–2011, demonstrated that the mean conception rate of ewes after AI and ET was 72% [15], suggesting that the reproductive potential of merino ewes may be negatively influenced by one or several underlying aetiological factors. AI and ET programs can be influenced by a number of environmental factors (e.g. climate, management practices, feed and water availability) and individual physiological traits (e.g. body condition score, disease status, age), which have the potential to impact upon the success of various methods applied in sheep breeding programs. Artificial insemination and ET are routinely used in most sheep stud enterprises and breeding businesses. Previous researches have also shown that varying influential factors, such as the sheep breed, age of the ewe, method (laparoscopic or cervical) of AI/ET, time between AI and synchronisation, type of semen (frozen or fresh) and the season of the year [16] can effect reproductive success. Artificial insemination and ET programs are advantageous to sheep production enterprises because they enable sheep breeders to enhance the genetic potential of their flock and minimize generation time, whilst being a cheaper, safer and more efficient alternative to natural mating.

The physiological stress response, through activation of the HPA axis is a traditional endocrine response to stress in vertebrates. Elevated secretion of glucocorticoids or cortisol under the influence of any physical or psychological stressor can negatively impact on reproductive hormone function. For example, glucocorticoids can minimize the secretion of gonadotrophin releasing hormone (GnRH) at the level of the hypothalamus and the hypophyseal portal system and, it can also reduce sensitivity and responsiveness of gonadotroph cells/receptors to GnRH [17]. Glucocorticoid suppression of reproductive function has been observed in sheep model during experimental exposure to infection reducing LH secretion, acute infusion of cortisol reduced sexual receptive behavior in ewe sheep [18]. In a study, [19] demonstrated that ewe sheep administered stress like concentrations of cortisol (intravenous delivery in right and left jugular veins at 0.1 mg/kg per hour 5 d before and 5 d after vaginal pessary removal) had significantly suppressed both oestrogen production and arrested follicular development during the late luteal and early follicular phase of oestrus cycle. [20] also reported similar results showing the effects of stress-like levels of cortisol on reproductive inhibition in sheep model. In a study conducted in sows, it was demonstrated that repeated stimulation of the HPA-axis using exogenous ACTH injections post-onset of estrus led to significant loss of oocytes or early embryos in the sows. Researchers concluded that a disturbance in oocyte maturation/quality could impair fertilization rate and embryo development [21]. Our preliminary results showed significant positive association between FGMs and reduced transferrable embryos in the merino ewes during both AI trials. In future studies, it will be worthwhile to investigate the effects of elevated FGMs during the estrus cycle, whether it associates with reduction in LH surge and positive feedback of estradiol for follicular/oocyte maturation and oocyte stimulation [22].

The significant interaction between FGMs and ewe body temperature as shown in our results is interesting as it suggests a plausible connection between thermal stress and stress endocrine response in the Australian merino ewes. Temperature humidity index is a widely used quantitative measure of thermal/heat stress in farm animals [23, 24]. For example, Vitali [25] showed that the chances of mortality in cattle increases when daily THI increases above 80. The THI index results were within severe thermal stress range (S1 Appendix) for the two AI trial period. It is assumed that elevated body temperature above normal level impedes certain reproductive processes that are crucial for embryo development. Heat stress is believed to reduce embryo production during AI/ET because the physiological and cellular aspects of reproductive function and early embryo development are disrupted. This increase in body temperature is due to the exposure to elevated ambient temperature, and by the physiological adaptations cells acquired to cope with thermal stress [26]. The reduction in the percentage of transferable embryos during heat stress can be attributed to a decrease in superovulatory response, lower fertilization rate and lower embryo quality [26]. In our study, ewes that had high rectal temperatures had significantly higher mean FCM concentration and lower mean percentage of transferable embryos compared to ewes that had lower rectal temperatures (see Table 1). This result suggests a plausible link between heat stress, physiological stress and reduced fertility in donor merino ewes.

Heat stress is postulated to affect folliculogenesis as it has been shown to supress follicular development, reduce 17β oestradiol concentration in follicular fluid and prevent the dominant ovarian follicle from exerting dominance [27, 28] in cattle. Oocyte competence is believed to be compromised by maternal thermal stress, whereby impaired folliculogenesis can result in the ovulation of aged follicles that contain oocytes with reduced competence that are unable to develop properly after fertilisation [26, 29]. In cattle that had undergone heat stress prior to slaughter, oocytes were harvested from collected ovaries and had undergone in vitro fertilization. The resulting embryos were found to have reduced competence to develop to the blastocyst stage [29].

Activation of the HPA-axis system *via* the physiological stress response, plays a crucial role in the regulation of reproduction in ewes [30]. Stress negatively affects reproductive physiology *via* the modulation of reproductive steroids GnRH, LH, progesterone and oestrogen levels within the hypothalamic-pituitary-gonadal axis (HPG-axis) system [31]. The stress response of the HPA-axis is elicited when a stimulus activates neurons within the hypothalamus of the brain, to synthesize and secrete corticotrophin-releasing hormone (CRH), which is the primary regulator of the HPA-axis [32]. Corticotrophin-releasing hormone is then released into the hypophyseal portal system and it is transported to the anterior pituitary gland, where it binds to specific receptors and induces the synthesis of the polypeptide tropic hormone adrenocorticotrophic hormone (ACTH) from the anterior pituitary and into the systemic circulation [33]. Adrenocorticotrophic hormone then targets the adrenal cortex of the adrenal glands and elicits the synthesis and release of glucocorticoid hormones such as cortisol [34]. Cortisol then acts on specific receptors throughout the peripheral tissues to regulate the physiological responses and behavioural changes associated with the stress response [33]. Cortisol is a glucocorticoid steroid hormone and is produced in the adrenal glands in response to stress and low plasma glucose levels [35]. When cortisol is released during the stress response, it functions to increase plasma glucose *via* gluconeogenesis, suppress the immune system and increase fat, protein and carbohydrate metabolism to aid in the stress response [36]. Elevated plasma cortisol is suggested as the primary aetiological factor driving failure of reproductive technology in sheep, as studies have shown that elevated cortisol levels from stress could arrest follicular development (Macfarlane et al., 2000), inhibit the pre-ovulatory LH surge [31] and can cause early embryonic loss and reduced implantation rate in sheep Dixon [37–39].

The reproductive efficacies of female sheep that are subjected to environmental conditions of high ambient temperature are decreased. Reproductive processes including embryogenesis are adversely affected by an unfavourable thermal environment. Heat stress may actually have a negative effect on fertility in livestock animals through modulation of the stress endocrine response [40]. One study found that cows that were exposed to heat stress had lower plasma cortisol concentrations, which was correlated with a decrease in conception rate and a lower percentage of palpable ovarian follicles on the day of oestrous however further studies are needed in various farmed livestock to validate these findings. It would have been useful for the results of this study if rectal temperature was taken from donor ewes after AI and prior to embryo flushing to determine if rectal temperature during early embryo development also affected embryo viability. However, this design would be difficult under standard industry practice as ewes were released into the paddock after the AI. Furthermore, comparing rectal temperature and FCM concentration of ewes over the estrus cycle, varying seasons (spring/summer and autumn/winter) during breeding programs would have significantly aided in validating the results of this study and could be a prospect for future research.

In conclusion, this study found that high rectal temperature and elevated FCM concentration was positively associated with a reduction in the percentage of transferable embryos recovered from donor Australian merino ewe sheep. The results of this study highlight that the efficacy of AI and ET breeding programs run in Australian summer can be influenced by physiological and environmental factors.

## Supporting information

S1 Appendix Sheep morphometric, physiological data, climate data and statistical analysis. (XLSX)
